# Impact of Ca_V_1.3 L-Type Calcium Channels on Arrhythmogenesis in Cancer

**DOI:** 10.3390/ijms27135663

**Published:** 2026-06-23

**Authors:** Lianlen Joy Go Distor, Yvonne Sleiman, Jean-Baptiste Reisqs, Vamsi Krishna Murthy Ginjupalli, Michael Cupelli, Mohamed Boutjdir

**Affiliations:** 1 Cardiovascular Research Program, VA New York Harbor Healthcare System, New York, NY 11209, USA; lianlenjoydistor@gmail.com (L.J.G.D.); yvonne_sleiman@hotmail.com (Y.S.); jeanbaptiste.reisqs@gmail.com (J.-B.R.); vamsikrishna.ginjupalli@va.gov (V.K.M.G.); michael.cupelli@va.gov (M.C.); 2 Department of Medicine, Cell Biology and Pharmacology, State University of New York Downstate Health Sciences University, New York, NY 11203, USA; 3 Department of Medicine, New York University Grossman School of Medicine, New York, NY 10016, USA

**Keywords:** arrhythmia, cardio-oncology, cardiotoxicity, cardiovascular disease, Ca_V_1.3 L-type calcium channel

## Abstract

Cardiovascular disease and cancer remain the leading causes of death worldwide. Although numerous cancer therapies have improved survival rates, they also increase the risk of cardiomyopathy, heart failure, and arrhythmias. These cardiovascular complications can limit treatment options and adversely affect the long-term quality of life of cancer survivors. Ca_V_1.3, an L-type calcium channel encoded by *CACNA1D*, emerges as a central molecular mediator linking cardiovascular disease and cancer. It regulates calcium entry into cardiomyocytes and contributes to sinoatrial pacemaking and atrioventricular conduction. It also contributes to proliferation, migration, and therapy resistance in several cancers. Chemotherapy-induced oxidative stress, inflammatory signaling, hypoxia, and transcriptional changes can modulate the expression, gating, splicing, and trafficking of Ca_V_1.3 channels. All these changes destabilize diastolic depolarization and impair conduction, thereby promoting arrhythmias in cancer patients. This review focuses on Ca_V_1.3 biology in cardio-oncology, along with the mechanisms of chemotherapy-induced cardiotoxicity. It outlines the role of Ca_V_1.3 as a key mediator linking cancer therapies to subsequent nodal dysfunction and increased arrhythmia susceptibility. It also expands on how patient-specific induced pluripotent stem cell-derived cardiomyocytes can model Ca_V_1.3 dysregulation as well as support the development of targeted therapies. We propose that Ca_V_1.3 represents a mechanistic bridge linking cancer therapy, calcium signaling, and cardiac electrophysiology, and that elucidating its pathophysiology may guide the design of targeted strategies in cardio-oncology.

## 1. Introduction

**Figure 1:**
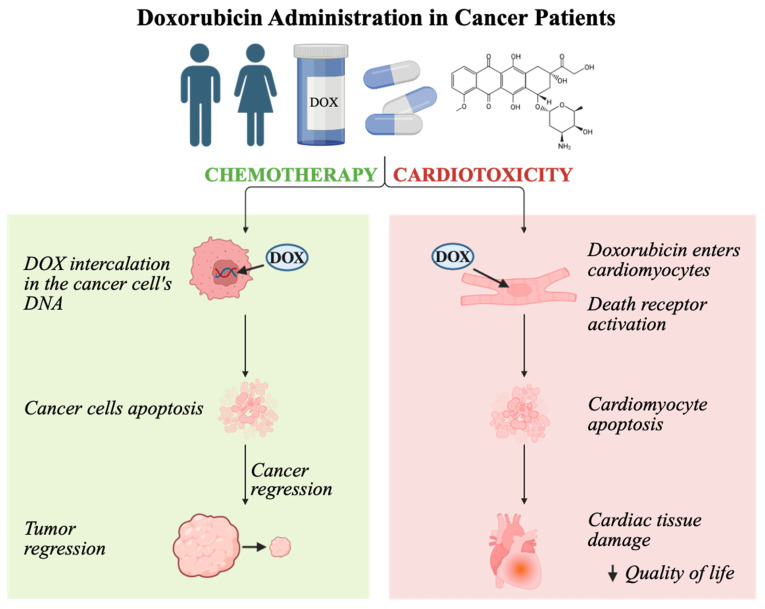
Therapeutic Action and Cardiotoxic Side Effects of Doxorubicin (DOX). (**Left**) DOX acts as an anticancer drug by DNA intercalation in cancer cells. This leads to apoptosis and tumor regression. (**Right**) At the same time, DOX can enter the cardiomyocytes, activate death receptor pathways, and induce apoptosis. This results in cardiac tissue damage and reduced quality of life.

Cardiovascular disease (CVD) and cancer are the leading causes of death worldwide [1,2,3,4]. Early detection and improved therapies have increased survival rates for both diseases, but cardiac complications induced by cancer treatments are still a major concern [5,6]. Across oncology, patients receiving chemotherapy, targeted therapies, or immunotherapies exhibit higher rates of arrhythmias and heart failure compared with both pre-treatment cancer patients and the general population [7]. Atrial fibrillation (AF) is the most common rhythm disturbance among both cancer patients and cancer survivors, occurring in the presence or absence of cardiotoxic cancer therapies [8]. Additionally, cardiac dysfunction such as heart failure, cardiomyopathy, arrhythmias, and coronary artery disease can appear during therapy or many years after cancer treatment, and these complications limit future cancer treatment options and significantly reduce quality of life [9,10] (Figure 1).

Ca_V_1.3, an L-type calcium channel (LTCC) encoded by the *CACNA1D* gene, represents a central mediator in both cardiac and cancer-related processes. It plays a critical role in cardiac pacemaking and atrioventricular (AV) conduction, while also contributing to tumor progression across multiple cancer types [11]. Ca_V_1.3 is sensitive to oxidative stress, mitochondrial dysfunction, and transcriptional alterations, pathways that are frequently activated by chemotherapy [12,13]. Given its dual role in both cardiology and oncology, Ca_V_1.3 may represent a critical mechanistic link between cancer therapy and cardiac electrophysiology.

This review explores the role of Ca_V_1.3 in linking chemotherapy to arrhythmias in cancer patients, highlighting its biological properties, its contribution to oncology-related cardiotoxicity, and emerging induced pluripotent stem cell (iPSC)-based platforms to study mechanisms and guide preventive therapies.

## 2. Ca_V_1.3 as a Key Player

### 2.1. Ca_V_1.3 in Physiological and Arrhythmogenic Cardiac Tissue

Voltage-Gated Calcium Channels (VGCCs) are important for electrical conduction and contraction in the heart because they regulate calcium (Ca^2+^) entry during membrane depolarization [14]. VGCCs are multiprotein complexes that contain α_1_, α_2_δ, β, and γ subunits, and the α_1_ subunit forms the ion-conducting pore, which determines the channel’s biophysical properties [11]. VGCCs are grouped into three major families based on their α_1_ subunit [11,15]. The Ca_V_1 family (Ca_V_1.1, Ca_V_1.2, Ca_V_1.3, and Ca_V_1.4), also known as LTCCs, produces long-lasting Ca^2+^ currents that support excitation-contraction coupling (ECC) [11,16]. The Ca_V_2 family (Ca_V_2.1–2.3) generates high-voltage-activated Ca^2+^ currents that mediate neurotransmission. The Ca_V_3 family (Ca_V_3.1–3.3) mediates low-voltage-activated T-type currents and lacks auxiliary subunits.

**Figure 2:**
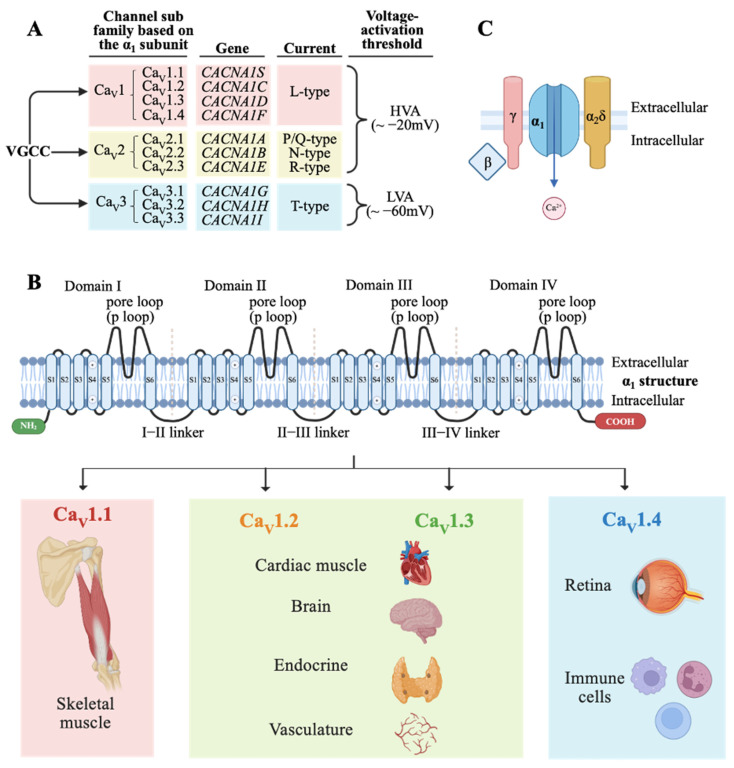
Structural and functional organization of voltage-gated calcium channels across tissues. (**A**) Classification of voltage-gated calcium channels (VGCCs) based on the pore-forming α_1_ subunit, corresponding human genes, current types, and voltage-activation thresholds. The Ca_V_1 family (Ca_V_1.1–Ca_V_1.4) mediates L-type currents, the Ca_V_2 family (Ca_V_2.1–Ca_V_2.3) mediates P/Q-, N-, and R-type currents, and the Ca_V_3 family (Ca_V_3.1–Ca_V_3.3) mediates T-type currents. High-voltage-activated (HVA) and low-voltage-activated (LVA) channel families are indicated. (**B**) Topology of the α_1_ subunit. The α_1_ subunit contains four homologous domains (I–IV), each composed of six transmembrane segments (S1–S6). The S1–S4 segments form the voltage-sensing domain, whereas the S5–S6 segments and pore (P) loops form the calcium-selective pore. L-type calcium channel isoforms display tissue-specific expression profiles, with Ca_V_1.1 primarily expressed in skeletal muscle; Ca_V_1.2 and Ca_V_1.3 are expressed in cardiac tissue, brain, endocrine organs, and vasculature; and Ca_V_1.4 is predominantly expressed in the retina and immune cells. (**C**) Schematic representation of the VGCC complex showing the pore-forming α_1_ subunit together with the auxiliary β, α_2_δ, and γ subunits, which regulate channel trafficking, membrane expression, and electrophysiological properties. Abbreviations: VGCC, voltage-gated calcium channel; HVA, high-voltage-activated; LVA, low-voltage-activated.

Structurally, the α_1_ subunit contains four homologous domains (I–IV), and each domain has six transmembrane helices (S1–S6) [15]. The S1-S4 segments form the voltage-sensing domain, and the S5–S6 segments form the channel pore and the Ca^2+^-selective filter [17]. Channel families also differ by activation threshold. Low-voltage-activated channels, such as T-type channels, activate near −60 mV [18]. High-voltage-activated channels, including LTCCs, activate near −40 mV [19,20] (Figure 2).

Each LTCC subtype is expressed in distinct tissues. Ca_V_1.1 is found in skeletal muscle [21,22]. Ca_V_1.2 and Ca_V_1.3 are present in the brain, heart, endocrine organs, and vasculature [23,24]. Ca_V_1.4 is limited to the retina and immune cells [25,26,27]. In the heart, Ca_V_1.2 is the dominant subtype in the ventricular myocardium. Ca_V_1.3 supports pacemaker activity in the sinoatrial (SA) node and ensures reliable conduction through the AV node because it activates at more negative membrane potentials [28,29]. The relatively hyperpolarized activation threshold of Cav1.3 channels facilitates the spontaneous diastolic depolarization phase within SA node cells, thereby serving as a primary determinant of cardiac chronotropy [30,31,32].

Ca_V_1.3 is expressed in the atria and pacemaker regions of the heart, where it supports diastolic depolarization and conduction [33,34]. Ca_V_1.3 expression is controlled through developmental, transcriptional, post-transcriptional, and post-translational mechanisms, and these regulatory processes differ between cardiac tissue and cancer tissue [12]. In the heart, these regulatory mechanisms influence pacemaking and conduction, whereas in tumors, they may contribute to proliferation, migration, and/or therapy resistance [35,36,37,38].

During cardiac development, Ca_V_1.3 is differentially expressed. Fetal and neonatal hearts contain Ca_V_1.3 in both supraventricular tissues and ventricles [39]. As the heart matures, Ca_V_1.3 becomes restricted to atrial and nodal regions and is no longer detected in the adult ventricular myocardium [39]. In human heart failure, Ca_V_1.3 reappears in the adult ventricles [40]. This re-expression may support Ca^2+^ entry and partially compensate for impaired ECC [40]. Similarly, Ca_V_1.3 has also been re-expressed in an adult murine heart failure model [41]. These developmental changes show that Ca_V_1.3 expression is responsive to physiological demand as well as pathological stress.

Transcriptional mechanisms also shape Ca_V_1.3 function. The C-terminus of the channel can translocate to the nucleus of cardiomyocytes, where it regulates the transcription of Ca^2+^-activated potassium channels (SK2) and upregulates expression of its own *CACNA1D* gene [12,42].

Alternative splicing adds another layer of control. The C-terminal region of Ca_V_1.3 undergoes extensive splicing, producing variants with distinct electrophysiological properties and drug sensitivities [43]. One major splice form (commonly referred to as the short isoform) lacks the C-terminal automodulatory domain. This domain normally interacts with calmodulin at the IQ motif to regulate Ca^2+^-dependent inactivation. Loss of the domain alters channel gating and Ca^2+^ sensitivity [43,44,45]. Work in inner hair cells first revealed that long C-terminal isoforms exhibit slow inactivation and high sensitivity to dihydropyridines such as nifedipine, whereas short isoforms inactivate rapidly and display reduced drug sensitivity. These observations demonstrate how splicing may contribute to tissue-specific channel behavior and differences in responsiveness to Ca^2+^ channel blockers.

Post-translational modifications also modulate Ca_V_1.3. In the heart, phosphorylation by protein kinase A (PKA) through the cyclic adenosine monophosphate (cAMP) pathway enhances Ca_V_1.3 activity at serine residues 1743 and 1816, strengthening pacemaker function in SA and atrial tissue, whereas protein kinase C (PKC)-dependent phosphorylation inhibits Ca_V_1.3, with an essential site identified at serine 81 in the N-terminal domain [34,46,47,48]. Sustained phosphorylation at serine 1475 by Ca^2+^/calmodulin-dependent protein kinase II (CaMKII) decreases current density [49]. The contribution of these kinase pathways to cancer is still not clear [50]. Ubiquitination regulates Ca_V_1.3 protein stability and trafficking, though its role in tumor biology is not yet fully defined [51,52]. Protein–protein interactions also add to the regulation. Calreticulin suppresses Ca_V_1.3 surface expression, and the auxiliary subunits β, α_2_δ, and γ are required for proper channel assembly, trafficking, and modulation [14]. The β_2_ subunit couples Ca_V_1.3 to big potassium (BK) channels, which shape action potential firing by accelerating repolarization in suprachiasmatic nucleus neurons [53]. This close functional coupling allows Ca_V_1.3-mediated Ca^2+^ entry to influence firing dynamics directly by activating BK-type K^+^ channels in the same tissue [54].

Pathogenic variants in *CACNA1D* are strongly associated with SA node dysfunction and AF. In animal models, Ca_V_1.3 knockout mice display sinus bradycardia, first-, second-, and third-degree AV block, and increased susceptibility to AF, reflecting impaired automaticity and disrupted AV conduction [33,48,55,56]. These findings demonstrate that Ca_V_1.3 function is required for stable pacing and reliable impulse propagation through nodal tissue.

During ECC, Ca_V_1.3 contributes to the initial Ca^2+^ influx that triggers downstream Ca^2+^ release [34]. Variants in *CACNA1D* can therefore alter intracellular Ca^2+^ handling and create arrhythmogenic conditions. For example, a study identified a heterozygous *CACNA1D* variant (p.Arg930His) in a family with sinus node dysfunction, epilepsy, and Attention Deficit Hyperactivity Disorder [57]. Functional studies showed loss of function in the long cardiac isoform of Ca_V_1.3, consistent with impaired automaticity and hence sinus node dysfunction. Conversely, Limpitikul and collaborators in 2016 reported that the Ca_V_1.3-A760G rat variant (human equivalent A749G) increased channel opening during inactivation and enhanced Ca^2+^ entry [58]. This gain-of-function effect highlights that *CACNA1D* variants can cause either bradyarrhythmia or tachyarrhythmia, depending on whether channel activity is decreased or increased. Combined with functional and genetic data, these observations identify Ca_V_1.3 as a central determinant of nodal electrophysiology, whose disruption leads to clinically relevant arrhythmias.

In addition to disease-associated *CACNA1D* variants, the functional consequences of Ca_V_1.3 dysregulation are influenced by its auxiliary subunits. The β and α_2_δ subunits are required for proper channel trafficking, membrane expression, and gating properties, whereas alterations in subunit composition can modify Ca^2+^ current (I_Ca,L_) density and channel availability [14,16]. Because nodal automaticity depends on tightly regulated Ca_V_1.3-mediated Ca^2+^ influx, disturbances in auxiliary subunit expression may impair diastolic depolarization and AV conduction. Furthermore, oxidative stress, inflammation, and remodeling pathways activated during cancer therapy may alter channel-subunit interactions, potentially contributing to nodal dysfunction and arrhythmogenesis. Although the specific roles of Ca_V_1.3 auxiliary subunits in cardio-oncology remain incompletely defined, their established importance in channel regulation suggests that they may represent additional modulators of cancer-associated electrical remodeling.

### 2.2. Ca_V_1.3 in Cancer Tissue

Ca_V_1.3 is increasingly recognized as a relevant ion channel in oncology, as it exhibits differential expression across multiple tumor types and demonstrates tissue-specific dysregulation [36,59]. Altered *CACNA1D* expression has been reported in several malignancies, including prostate, breast, ovarian, and endometrial cancers, where Ca_V_1.3 is upregulated [35,36,37,38,59,60]. Importantly, the role of Ca_V_1.3 is not uniform across cancers. Variations in expression levels, alternative splicing patterns, and subcellular localization among cancer types contribute to distinct functional outcomes, thereby shaping the channel’s context-dependent impact on tumor biology. Studies indicate that Ca_V_1.3 expression is influenced by tumor-associated signaling pathways such as androgen receptor signaling, hypoxia-responsive pathways, inflammatory signaling, and non-coding RNA (ncRNAs) networks [35,59,60,61,62]. These findings are particularly relevant to cardio-oncology because stress-responsive pathways that regulate Ca_V_1.3 expression are also activated during cancer treatment, suggesting that chemotherapy-induced alterations in Ca^2+^ homeostasis, oxidative stress, and inflammatory signaling may modulate its expression.

Regulation in cancer tissue differs substantially from regulation in the heart. In prostate cancer, androgen receptor signaling increases Ca_V_1.3 expression, and the *TMPRSS2-ERG* fusion gene activates *CACNA1D* transcription, contributing to tumor survival and resistance to androgen deprivation therapy [59,63]. Hypoxia, a hallmark of many solid tumors, differentially modulates Ca_V_1.3 expression depending on the cellular context, decreasing its expression in neuroblastoma while promoting its upregulation in superficial bladder cancer via increased *CACNA1D* transcription [35,64]. ncRNAs also contribute to the regulation of Ca_V_1.3 expression. In gastric cancer models, transfer RNA (tRNA)-derived small RNAs (tsRNAs), a type of ncRNA, have been reported to be downregulated, and restoration of their expression suppresses cellular proliferation, suggesting a potential tumor-suppressive role [62,65]. Additionally, elevated expression of the tRNA-derived fragment (tRF) tRF-Val-CAC-016 has been associated with suppression of gastric carcinoma cell proliferation through modulation of *CACNA1D*-mediated MAPK signaling [62].

These regulatory mechanisms further highlight the influence of oncogenic signaling pathways on Ca_V_1.3 expression. Consequently, selective inhibition of Ca_V_1.3 activity may reduce tumor growth and metastatic potential in a cancer type-dependent manner. In the heart, reduced Ca_V_1.3 activity or expression in SA and AV node cells could normalize diastolic depolarization and pacemaker activity, thereby attenuating node-dependent arrhythmias. However, the potential therapeutic implications of Ca_V_1.3 modulation have not yet been evaluated in cancer-specific models [33]. Additional investigations in cancer patients and preclinical animal models are required to establish whether cancer directly affects cardiac Ca_V_1.3 trafficking. Current knowledge of Ca_V_1.3 trafficking mechanisms is derived largely from non-cancer cardiac disease models, particularly AF and heart failure [34].

Oxidative stress, inflammation, autonomic dysregulation, mitochondrial dysfunction, and electrical remodeling are of particular interest because they modulate molecular pathways involved in the regulation of Ca_V_1.3 expression, trafficking, phosphorylation, and channel function. Systemic inflammation can influence Ca^2+^-dependent transcriptional regulators of *CACNA1D*, hypoxia can alter intracellular Ca^2+^ homeostasis and Ca_V_1.3 expression, and neurohormonal activation can modify channel phosphorylation and trafficking [38]. In parallel, anthracycline-induced oxidative stress and mitochondrial dysfunction may promote remodeling of Ca_V_1.3-dependent signaling pathways [66].

**Figure 3:**
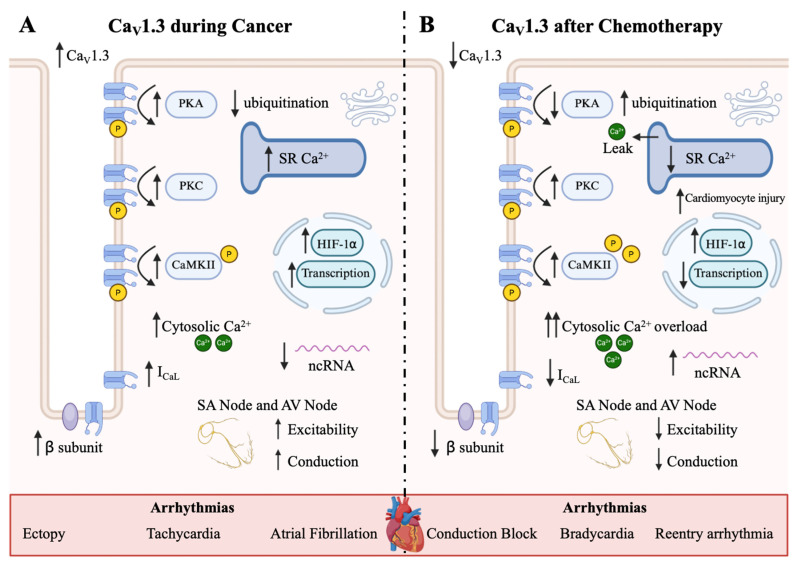
Proposed mechanisms by which cancer and chemotherapy may differentially regulate Ca_V_1.3 channels and contribute to arrhythmogenesis. (**A**) During cancer, Ca_V_1.3 expression and function could be enhanced through increased PKA-, PKC-, and CaMKII-dependent phosphorylation, reduced ubiquitination, activation of HIF-1α signaling, increased *CACNA1D* transcription, decreased non-coding RNA (ncRNA), enhanced sarcoplasmic reticulum (SR) Ca^2+^ load, increased β-subunit availability, and increased basal cytosolic Ca^2+^. These changes could increase L-type Ca^2+^ current (I_Ca,L_), promote sinoatrial (SA) and atrioventricular (AV) nodal excitability and conduction, and increase susceptibility to ectopy, tachycardia, and atrial fibrillation. (**B**) In contrast, chemotherapy-induced stress may reduce Ca_V_1.3 expression and activity through decreased PKA signaling, increased ubiquitination, increase in HIF-1α signaling and cardiomyocyte injury, decreased *CACNA1D* transcription, increased ncRNA, reduced SR Ca^2+^ content, increased basal cytosolic Ca^2+^ overload, and loss of β-subunit support, resulting in decreased I_Ca,L_, impaired SA and AV nodal function, reduced excitability and conduction, and an increased risk of conduction block, bradycardia, and reentrant arrhythmias.

Patients with cancer can develop cardiac arrhythmias even before the initiation of chemotherapy, including ectopy, tachycardia, and AF [67]. Tumor-associated inflammation, oxidative stress, and hypoxia may contribute to this electrical remodeling by altering Ca^2+^-handling pathways and ion channel regulation [68]. In the cancer setting, Ca_V_1.3 is proposed to be upregulated through increased HIF-1α signaling, elevated *CACNA1D* transcription and mRNA expression, enhanced PKA-, and CaMKII-dependent phosphorylation, reduced ubiquitination-mediated degradation, increased β-subunit support, increased basal cytosolic Ca^2+^ and decreased ncRNAs [11,37]. Enhanced HIF-1α signaling could promote *CACNA1D* transcription, resulting in increased Ca_V_1.3 expression [38]. Cellular stress, particularly through elevated catecholamine and cytokine levels, activates β-adrenergic signaling, leading to increased cAMP production and PKA activity. PKA-mediated phosphorylation of Ca_V_1.3 channels is likely to increase their open probability, thereby enhancing Ca^2+^ influx and I_Ca,L_ in nodal cells, which may increase automaticity and promote arrhythmogenesis. Stress-related signaling can also activate PKC isoforms, which phosphorylate Ca_V_1.3 channels and might alter channel gating by decreasing their open probability and I_Ca,L_ in an isoform-dependent manner. In addition, increased CaMKII activity can elevate intracellular Ca^2+^ levels and enhance nodal excitability [69]. Increased support from auxiliary β-subunits might further augment Ca_V_1.3 channel function and I_Ca,L_. Conversely, reduced ubiquitin-mediated degradation stabilizes Ca_V_1.3 channels, promoting their trafficking and surface expression and thereby increasing nodal excitability. Finally, downregulation of ncRNAs that normally repress *CACNA1D* expression may enhance transcription of the channel, leading to increased Ca_V_1.3 expression and membrane availability. Overall, these mechanisms could possibly increase channel surface expression and activity, augment I_Ca,L_, enhance sarcoplasmic reticulum (SR) Ca^2+^ load, and promote intracellular Ca^2+^ signaling. Because Ca_V_1.3 is highly expressed in the SA and AV nodes, cancer-associated Ca_V_1.3 upregulation may enhance nodal excitability and conduction, leading to ectopy, tachycardia and AF [34] (Figure 3).

In contrast, chemotherapy-induced oxidative injury and cellular stress could impair Ca_V_1.3 function through multiple mechanisms, including elevated HIF-1α signaling, reduced PKA activity, enhanced ubiquitin-mediated channel degradation, loss of β-subunit support, increased ncRNAs, increased PKC activation, elevated basal cytosolic Ca^2+^ levels, and depletion of SR Ca^2+^ stores [11]. Chemotherapy has been shown to increase HIF-1α signaling [70,71], which in the setting of cardiotoxicity is associated with cardiomyocyte injury, inflammation, and adverse cellular remodeling that may indirectly impair Ca_V_1.3 regulation. In parallel, cardiomyocyte damage could attenuate β-adrenergic/PKA signaling, reducing Ca_V_1.3 phosphorylation and I_Ca,L_, thereby slowing nodal activity. Enhanced ubiquitination likely promotes proteasomal degradation of Ca_V_1.3 channels, leading to a decrease in Ca_V_1.3 protein stability, while the loss of β-subunit support may impair channel trafficking and membrane localization, leading to a decrease in Ca_V_1.3 surface expression. Increased expression of ncRNAs can further suppress *CACNA1D* expression and reduce channel availability at the cell surface. Oxidative stress-induced PKC activation may alter channel gating and decrease its open probability and I_Ca,L_, whereas SR Ca^2+^ depletion and cytosolic Ca^2+^ overload due to SR Ca^2+^ leak would further disrupt Ca^2+^ homeostasis and nodal excitability. Collectively, these changes may reduce Ca_V_1.3 surface expression and I_Ca,L_, thereby impairing Ca^2+^-dependent electrical activity. Consequently, chemotherapy-associated Ca_V_1.3 dysregulation could impair pacemaker function and impulse propagation, contributing to bradyarrhythmias, conduction blocks, and other forms of SA and AV nodal dysfunction [72,73] (Figure 3). However, despite growing evidence implicating Ca_V_1.3 in cardiac electrophysiology, its specific role in the development of cancer-associated arrhythmias remains incompletely understood and requires further investigation. Moreover, the electrophysiological consequences of Ca_V_1.3 dysregulation may vary among patients depending on factors such as age, comorbidities, genetic background, and treatment exposure. Interestingly, in a guinea pig model of doxorubicin (DOX)-induced cardiotoxicity, I_Ca,L_ was not significantly altered despite evidence of impaired calcium-induced calcium release resulting from increased SR Ca^2+^ leak. This observation highlights the complexity of Ca^2+^ dysregulation in anthracycline-induced cardiotoxicity and warrants further investigation into the contribution of LTCC [74,75].

Given that Ca_V_1.3 is expressed in both tumor cells and the heart, treatment-induced alterations in channel regulation may have consequences beyond tumor control and could contribute to electrical disturbances within the cardiovascular system [76]. Consequently, pro-inflammatory cytokines such as interleukin-6 and tumor necrosis factor-alpha contribute to promoting myocardial fibrosis, ion channel dysfunction, and autonomic dysregulation, thereby creating a substrate for promoting arrhythmogenesis [77]. Collectively, these findings suggest that molecular pathways regulating Ca_V_1.3 in cancer may intersect with those activated by chemotherapy-induced cellular stress, providing a potential mechanistic link between cancer treatment, altered cardiac Ca_V_1.3 signaling, and increased arrhythmia susceptibility.

Aside from Ca_V_1.3, other members of the Ca_V_1 family could also contribute to cancer-associated cardiovascular dysfunction. Ca_V_1.2, being the predominant LTCC in ventricular myocardium, plays a central role in ECC; therefore, chemotherapy-induced alterations in Ca_V_1.2 function may contribute to contractile dysfunction and ventricular arrhythmias [78]. In contrast, Ca_V_1.1 and Ca_V_1.4 are primarily expressed in skeletal muscle and retinal tissue, respectively, and their roles in cardio-oncology remain poorly defined. Nonetheless, Ca_V_1.3 is uniquely positioned at the intersection of cancer biology and cardiac electrophysiology because of its established roles in tumor-associated signaling, SA and AV nodal function, and arrhythmogenesis.

Cancer therapies frequently destabilize cardiac electrophysiology, resulting in a high incidence of arrhythmias in oncology patients [79]. These include atrial and ventricular arrhythmias, premature beats, conduction disturbances, and bradyarrhythmias, with AF being the most common [68,80]. While AF is also common in the general population, its incidence is significantly higher in cancer patients and often develops earlier and progresses more rapidly [68]. Although multiple mechanisms contribute to arrhythmogenesis in cancer, many converge on pathways that regulate intracellular Ca^2+^ handling and nodal electrophysiology.

**Figure 4:**
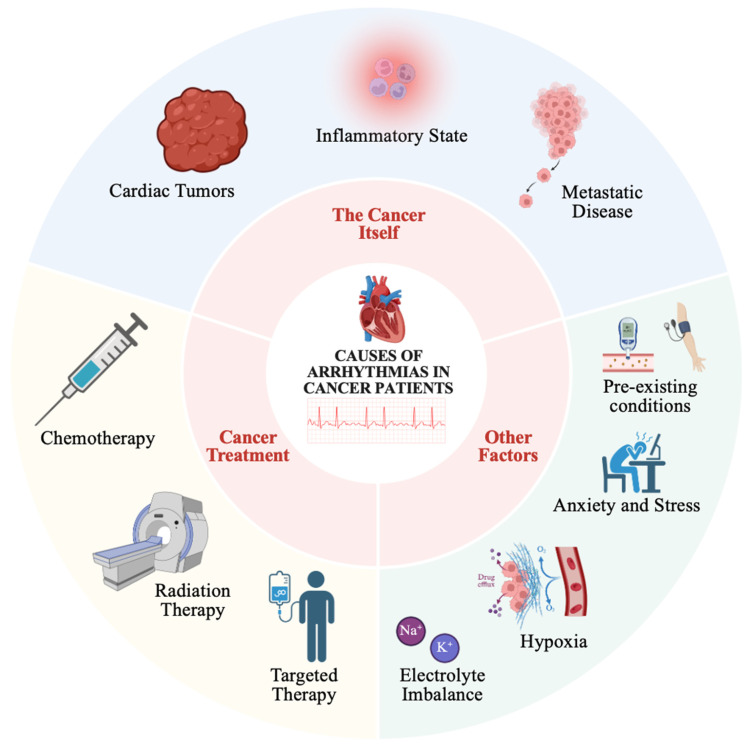
Causes of Arrhythmias in Cancer Patients. Arrhythmias in cancer patients may result from the cancer itself, anticancer therapies, or other contributing factors.

Cancer-associated arrhythmias result from a complex interplay of tumor-related, treatment-related, and systemic factors (Figure 4). For example, cardiac tumors can directly disrupt electrical conduction pathways, resulting in impaired impulse propagation and conduction block [81,82]. Although cardiac tumors may contribute to arrhythmogenesis, it is important to note that approximately 90% of primary cardiac tumors are benign, whereas only 10% are malignant [83]. In addition, anthracyclines, tyrosine kinase inhibitors, radiation therapy, immune checkpoint inhibitors, and other targeted anticancer therapies can induce oxidative stress, inflammation, autonomic dysfunction, mitochondrial impairment, and electrical remodeling, all of which contribute to arrhythmia development [84,85,86,87,88]. Systemic consequences of malignancy, including chronic inflammatory signaling, hypoxia, and neurohormonal activation, could further destabilize cardiac electrophysiology and increase arrhythmia susceptibility [68,89,90].

Other than Ca_V_1.3, several genes have been implicated in both cancer and CVD, and these are highlighted in Table 1. For example, the *DNMT3A* gene encodes a DNA methyltransferase protein that regulates hematopoiesis through epigenetic mechanisms and functions as a tumor suppressor. Mutations in *DNMT3A* have been linked to inflammatory processes that contribute to both cancer and cardiovascular pathology [91,92]. Similarly, *TET2* encodes a transcriptional regulator and tumor suppressor that catalyzes the conversion of 5-methylcytosine to 5-hydroxymethylcytosine. Like *DNMT3A*, *TET2* is involved in inflammatory pathways associated with the development of both cancer and CVD [93].

Given the apparent sensitivity of Ca_V_1.3 to stress-related signaling pathways activated in cancer, anticancer therapies that engage these pathways may perturb its regulation. Understanding the mechanisms of action of major chemotherapeutic agents could therefore provide insight into how cancer treatment alters Ca_V_1.3 signaling and contributes to arrhythmia susceptibility.

## 3. Chemotherapy Drugs and Cardiotoxic Effects

### 3.1. Chemotherapy Drug Classes and Their Cardiotoxic Effects

Chemotherapeutic agents are central to modern cancer treatment; however, many target or indirectly perturb signaling pathways that regulate Ca_V_1.3 [11]. Major drug classes, including anthracyclines, antimetabolites, BCL 2 inhibitors, hypomethylating agents, tyrosine kinase inhibitors, and antibody drug conjugates, are associated with cardiotoxicity mediated by mechanisms such as oxidative stress, mitochondrial dysfunction, activation of DNA damage responses, and disruption of intracellular Ca^2+^ homeostasis [85,110,111,112,113]. These stress pathways can modify Ca_V_1.3 transcription, gating, trafficking, or stability, creating a direct link between chemotherapy exposure and nodal electrical remodeling.

Anthracyclines such as DOX, daunorubicin, and epirubicin have the strongest known effects on Ca_V_1.3. They generate reactive oxygen species (ROS), injure mitochondria, and activate apoptotic signaling [114,115]. These processes alter Ca^2+^-dependent transcription, change the phosphorylation environment that controls Ca_V_1.3 gating, and disrupt channel trafficking. In nodal tissue, these changes may slow diastolic depolarization, weaken SA node automaticity, or impair AV node conduction. Vulnerability is heightened in children, the elderly, and patients with prior radiation or combination treatments, driven by the accelerated accumulation of oxidative and inflammatory stressors [84,85,116,117,118].

Antimetabolites such as 5-fluorouracil and capecitabine produce vasospasm, endothelial injury, and oxidative stress [112,119,120,121]. These mechanisms disturb intracellular Ca^2+^ homeostasis and activate signaling pathways that regulate *CACNA1D* (Ca_V_1.3) expression and trafficking. Patients with ischemic heart disease or thiopurine methyltransferase deficiency may experience stronger Ca_V_1.3 shifts (altered channel localization, stability, or functional properties) because their Ca^2+^-handling reserve is already compromised [122,123].

BCL-2 inhibitors and hypomethylating agents activate pro-apoptotic and mitochondrial stress pathways. Both influence Ca_V_1.3 by altering Ca^2+^-activated transcription and by changing post-translational regulation that determines channel stability. Tyrosine kinase inhibitors such as nilotinib, dasatinib, sorafenib, and ibrutinib modify signaling cascades that control ion channel trafficking. These drugs may shift Ca_V_1.3 localization in nodal tissue or disrupt its interaction with auxiliary subunits [110,124,125,126,127,128,129].

Antibody-drug conjugates such as gemtuzumab ozogamicin deliver highly cytotoxic agents that create oxidative stress, DNA damage, and mitochondrial dysfunction in cardiac cells [130,131,132,133]. These pathways can alter Ca_V_1.3 through ubiquitination, changes in auxiliary subunit interactions, or shifts in the balance between long and short Ca_V_1.3 isoforms. Older patients and those with relapsed or refractory acute myeloid leukemia have higher susceptibility because they begin treatment with high oxidative and inflammatory burdens.

Across these drug classes, chemotherapy modifies Ca_V_1.3 at multiple regulatory levels, including transcription and phosphorylation. These changes can alter nodal excitability and increase arrhythmia risk. This Ca_V_1.3-centered view provides the rationale for examining how chemotherapy remodels nodal electrophysiology and why certain therapies increase susceptibility to atrial and AV conduction abnormalities.

### 3.2. Mechanisms of DOX-Induced Cardiotoxicity

DOX is one of the most widely used anthracyclines and remains a core treatment for cancers such as pediatric acute lymphoblastic leukemia and acute myeloid leukemia [127,134]. Anthracyclines intercalate into DNA and inhibit topoisomerase II, which produces ROS, causes DNA damage, and triggers apoptosis in rapidly dividing cells [135,136]. Despite their effectiveness, anthracyclines cause cardiotoxicity [9,84]. Long-term survivors face an increased risk of cardiomyopathy, heart failure, and arrhythmias years after treatment [9,84].

DOX alters Ca^2+^-dependent transcription factors such as CREB and NFAT, which regulate *CACNA1D* expression. These Ca^2+^-sensitive transcription factors link DOX-induced Ca^2+^ signaling to transcriptional programs that can modulate *CACNA1D* expression and downstream excitability [60,61]. Different regulatory mechanisms may serve as the interface through which treatment-associated stress alters Ca_V_1.3 regulation. Anthracycline-induced oxidative stress, hypoxia, and inflammatory signaling may influence Ca_V_1.3 transcription, splicing, phosphorylation, and ubiquitination (Figure 3). Meanwhile, in mouse models of DOX-induced cardiotoxicity, DOX treatment increases CaMKII autophosphorylation at Thr287 and CaMKII oxidation, leading to persistently activated CaMKII and results in more phosphorylation of Ca_V_1.3, which lowers the I_Ca,L_ [137]. Additionally, CaMKII inhibition has been shown to prevent DOX-induced cardiac dysfunction and LTCC blockade with nifedipine suppresses DOX-induced CaMKII phosphorylation [138]. Notably, CaMKII inhibition has been shown to increase ubiquitin-specific protease 10 expression in DOX-treated mouse hearts, H9C2 cells and HL-1 cells [139]. Therefore, CaMKII activation promotes ubiquitination by inhibiting Ubiquitin Specific Peptidase 10 (USP10). This then can imply that Ca_V_1.3 is trafficked less. However, while PKA- and PKC-dependent regulation of LTCC is well established in cardiac tissue, direct evidence linking DOX exposure to Ca_V_1.3 phosphorylation through these pathways remains limited.

During ventricular remodeling, DOX has been shown to reactivate fetal ion channel gene expression patterns, which in some heart failure models is associated with increased Ca_V_1.3 expression in ventricular tissue [40,41]. Together, these findings indicate that DOX may influence Ca_V_1.3 through transcriptional, post-translational, and Ca^2+^-dependent regulatory pathways.

DOX causes cellular injury at the organelle level at multiple sites. In the nucleus, DOX produces DNA strand breaks and accelerates telomere shortening [140]. In mitochondria, DOX binds cardiolipin, accumulates within the inner mitochondrial membrane, and disrupts the electron transport chain. These changes lead to mitochondrial dysfunction and increased ROS production [141]. In the cytoplasm, DOX reduces Ca^2+^ reuptake into the endoplasmic reticulum, elevating cytoplasmic Ca^2+^ levels [142,143]. DOX also inhibits fatty acid transport into mitochondria, which reduces ATP availability and limits contractile reserve [144,145,146,147,148].

These mechanisms converge on pathways that critically regulate cardiac electrophysiology. Increased ROS generation and mitochondrial dysfunction destabilize nodal automaticity. Elevated cytosolic Ca^2+^ modifies Ca^2+^-dependent transcriptional signaling and alters ion channel inactivation kinetics. Reduced ATP availability compromises Na^+^/K^+^-ATPase activity, leading to perturbations in resting membrane potential. Collectively, these alterations contribute to DOX-associated AF, QT interval prolongation, conduction abnormalities, and diastolic dysfunction [149,150,151].

Since Ca_V_1.3 is a fundamental regulator of diastolic depolarization and AV conduction, any alterations in its expression, phosphorylation, or inactivation kinetics triggered by DOX are poised to directly undermine nodal excitability and cardiac rhythm stability.

### 3.3. Role of Ca_V_1.3 Dysregulation in DOX-Induced Stress

Ca_V_1.3 also participates in broader stress-response pathways that may contribute to anthracycline injury. In the context of DOX injury, Ca_V_1.3 dysregulation amplifies cellular dysfunction through three pathways. First, altered Ca_V_1.3 activity disrupts intracellular Ca^2+^ homeostasis and worsens oxidative and metabolic damage [152,153]. These changes may impair the ability of nodal cells to maintain stable pacemaker activity and increase vulnerability to abnormal impulse generation. Second, DOX-induced changes in Ca_V_1.3 expression can impair autophagy and cell survival pathways, reducing the cell’s ability to recover from chemotherapeutic injury. In nodal tissue, oxidative and metabolic stress may further compromise automaticity and conduction because pacemaker cells depend on precise Ca^2+^ handling for normal diastolic depolarization. Third, because Ca_V_1.3 is expressed in both the heart and the brain, its dysfunction influences neurocardiac signaling and autonomic regulation [76,154,155]. Altered sympathetic and parasympathetic input may destabilize SA node activity and AV conduction, thereby amplifying the arrhythmogenic consequences of direct cardiac Ca_V_1.3 dysfunction. These combined effects indicate that Ca_V_1.3 may contribute to cardiotoxicity both directly, through impaired Ca^2+^ handling in cardiomyocytes, abnormal diastolic depolarization, and conduction disturbances, and indirectly, through altered sympathetic and parasympathetic output.

## 4. Therapeutic Strategies and Future Directions

Ca_V_1.3 occupies a critical interface between cardiac electrophysiology and cancer-associated signaling, positioning it as a compelling therapeutic target. Its dysregulation is associated with arrhythmias, impaired stress responses, and poor cancer prognosis, and it also contributes to neuronal and metabolic signaling [76,156]. Several studies show that Ca^2+^ channel blockers produce mixed outcomes in oncology. Some inhibitors suppress tumor proliferation, while others increase migration or invasiveness [37]. These findings emphasize that nonselective modulation of LTCCs is risky and that precision in both drug design and delivery is essential.

Ca_V_1.3 represents an attractive therapeutic target because cancer cells frequently express distinct splice isoforms and exhibit altered post-translational channel modifications. These molecular signatures differ from the forms expressed in healthy SA and AV node tissue. Anthracycline exposure may also shift Ca_V_1.3 splicing and phosphorylation patterns in the heart. Identifying these disease-associated channel states represents a crucial step toward the design of selective Ca_V_1.3 inhibitors. Small molecules, peptides, or biologics designed to bind specific Ca_V_1.3 conformations could suppress oncogenic signaling while sparing Ca_V_1.3 currents that maintain normal pacemaking and autonomic regulation.

Ca^2+^ channel blockers such as nifedipine, verapamil, and diltiazem do not distinguish Ca_V_1.3 from Ca_V_1.2. Verapamil can reduce proliferation in some cancer models but can also increase metabolic stress in cardiomyocytes. Nifedipine inhibits breast cancer proliferation in some contexts but enhances migration in others [37]. These conflicting results show that Ca_V_1.3 requires targeted rather than broad LTCC inhibition.

Selective Ca_V_1.3 modulators paired with tissue-targeted delivery approaches may hold promise in terms of research. Nanoparticle-based carriers, antibody-guided delivery systems, and tumor-selective promoters could enable targeted delivery of chemotherapy to cancer cells expressing Ca_V_1.3, while minimizing exposure of cardiac tissue and reducing off-target cardiac toxicity. Gene therapy approaches present another possibility. Viral vectors carrying engineered *CACNA1D* variants could also correct pathological Ca_V_1.3 activity in remodeled nodal tissue [50,52].

The intersection between Ca_V_1.3 biology and anthracycline-induced cardiotoxicity has strong translational potential. Therapeutic strategies aimed at Ca_V_1.3 or its regulatory networks may offer a synergistic advantage, simultaneously enhancing oncological outcomes and mitigating chronic cardiotoxicity.

Human iPSC-CMs provide a platform to test these strategies, especially since technology now allows for differentiation of iPSCs into pacemaker-like, atrial-like and ventricular-like myocytes [157]. These cell-type-specific iPSC-CMs from patients who develop DOX-induced arrhythmias can reveal how DOX alters Ca_V_1.3 expression, splicing, and downstream signaling. These models support potential screening of Ca_V_1.3-selective modulators and protective adjunct therapies. They also offer a route for personalized cardiotoxicity prediction before treatment begins. Integrating human iPSC-based cell models with Ca_V_1.3-targeted therapeutic development could create strategies that correct pathological Ca_V_1.3 activity while preserving its essential roles in pacemaking, neuronal signaling, and endocrine function.

## 5. Research Tools for Cardio-Oncology

Cardio-oncology research requires models that represent both cardiac electrophysiology and cancer treatment responses. Because Ca_V_1.3 controls nodal pacemaking and changes during chemotherapy, the models used should capture its expression and gating at a minimum. The validity of the model is determined by its ability to preserve the molecular and functional properties of Ca_V_1.3.

Rodent models offer genetic tools, including *CACNA1D* knockout and knock-in lines, which allow direct investigation of Ca_V_1.3 in SA and AV nodes [158]. Given their high Ca_V_1.3 expression in nodal regions, these rodents provide an appropriate platform to examine how anthracyclines modulate nodal excitability and pacemaker function. Pig models of severe anthracycline injury show altered Ca^2+^ signaling and conduction slowing, which makes them suitable for studying Ca_V_1.3 remodeling with advanced imaging [159].

Zebrafish provide rapid genetic manipulation and optical mapping. They express *CACNA1D* orthologs in their AV conduction system [160]. Their transparent embryos allow direct visualization of conduction abnormalities after DOX exposure. Although zebrafish Ca_V_1.3 differs from the human channel, these models reveal conserved injury pathways and enable genetic screens for Ca_V_1.3 regulators.

Several research tools support these models. Patch-clamp electrophysiology measures Ca_V_1.3 currents, activation thresholds, and Ca^2+^-dependent inactivation directly [11,161,162,163]. Multi-omics approaches identify changes in *CACNA1D* transcription and post-translational modification after chemotherapy [161]. Echocardiography and cardiac magnetic resonance define structural and functional changes in vivo and provide endpoints for testing Ca_V_1.3-targeted therapies.

As mentioned above, hiPSCs are valuable for Ca_V_1.3 research. Patient-derived hiPSC cardiomyocytes from individuals who developed chemotherapy-induced arrhythmias maintain the underlying genetic and epigenetic determinants that modulate Ca_V_1.3 functional regulation [164]. These cells express Ca_V_1.3 and allow direct testing of how DOX alters Ca_V_1.3 and downstream Ca^2+^ signaling. They also reveal patient-specific vulnerabilities such as altered *CACNA1D* transcription, shifts in the ratio of long and short Ca_V_1.3 isoforms, or changes in Ca^2+^-dependent inactivation.

A recent study of pediatric anthracycline cardiotoxicity showed increased DOX susceptibility in patient-specific human iPSC-CMs due to downregulation of protective microRNAs [165]. Although that study did not examine Ca_V_1.3, its design supports future works that use patient-derived human iPSC-CMs to measure Ca_V_1.3 currents and identify Ca_V_1.3-dependent biomarkers of arrhythmia risk.

## 6. Conclusions

Cardio-oncology examines how cancer and its treatments affect the heart. Cancer therapies improve survival, but many damage the heart and increase the risk of arrhythmias and heart failure. These complications limit treatment choices and reduce the quality of life for survivors. Understanding their mechanisms is essential.

This review demonstrates the central role of Ca_V_1.3 in cardio-oncology toxicity. Ca_V_1.3 regulates pacemaking, conduction, Ca^2+^ entry, and cell stress responses. It also contributes to tumor biology. Anthracycline injury pathways converge on Ca_V_1.3, altering its expression, gating kinetics, and trafficking patterns. These perturbations disrupt nodal activity and increase arrhythmia risk, positioning Ca_V_1.3 as a pivotal mechanistic link between chemotherapy exposure and subsequent cardiac dysfunction.

Further investigation is essential to elucidate the precise molecular and cellular mechanisms by which chemotherapy modifies Ca_V_1.3. Ca_V_1.3 is a promising target for therapy and risk stratification in cardio-oncology. It provides a single framework that connects cancer treatment, cardiac electrophysiology, and stress biology. Focusing on Ca_V_1.3 could lead to biomarkers and interventions that reduce arrhythmias in cancer patients without compromising anticancer efficacy.

## Figures and Tables

**Table 1:** Gene variants associated with tumors and CVDs.

Gene Variants	Function of the Protein	Relation to Cancer and CVD	References
*DNMT3A*	A DNA methyltransferase that epigenetically regulates hematopoiesis and functions as a tumor suppressor.	Associated with an inflammatory response	[91,92]
*TET2*	A transcriptional regulator and tumor suppressor Facilitates the oxidation of 5-methylcytosine into 5-hydroxymethylcytosine	Associated with an inflammatory response	[93]
*DYRK*	DYRK1A isoform: Phosphorylates TNF receptor-associated factor 3 (TRAF3), leading to activation of the non-canonical NF-κB signaling pathway.	Promotes the development of acute lymphoblastic leukemia (ALL). In myocardial infarction (MI) mouse models, *DYRK1A* knockdown triggers cardiomyocyte cell-cycle reactivation and upregulation of proliferation-related genes. Knockdown also increases epigenetic markers H3K4me3 and H3K27ac, indicating a role in regulating chromatin states during cardiac repair.	[94,95]
	DYRK1B isoform: Regulates expression of mitochondrial electron transport chain complexes, supporting oxidative phosphorylation and energy metabolism.	Overexpression in cancer cells promotes cell proliferation and resistance to chemotherapy. Facilitates G0/G1 to S phase progression and enhances the expression of antioxidant genes.	[96,97]
*BRCA1/2*	Play a key role in DNA damage repair	Hereditary breast and ovarian cancers and have been found to be related to CVD. May increase vulnerability to myocardial injury following exposure to radiotherapy or chemotherapy (example, DOX)	[98,99,100]
*JAK2*	Encodes a Janus kinase involved in cytokine signaling, which influences inflammation, hematopoiesis, and cell proliferation.	*JAK2* V617F is the well-known variant that leads to myeloproliferative neoplasms (MPNs). As for CVD, clonal hematopoiesis with *JAK2* variants is often associated with an increased risk of CVD. Patients with *JAK2* variants have a ~2x increase in thrombotic event risk and as well as higher cardiovascular mortality.	[101,102,103,104]
*TTN*	Encodes titin	Truncated *TTN* variants are a major cause of dilated cardiomyopathy and other cardiac muscle diseases. Associated with heart failure. *TTN* variants occur frequently across cancer types such as liver, lung, and endometrial cancers.	[105,106,107,108,109]

## Data Availability

No new data were created or analyzed in this study. Data sharing is not applicable to this article.

## Abbreviations

ALL	Acute lymphoblastic leukemia
AV	Atrioventricular
CM(s)	Cardiomyocyte(s)
CVD	Cardiovascular disease
DOX	Doxorubicin
hiPSC-CM(s)	Human induced pluripotent stem cell-derived cardiomyocyte(s)
iPSC(s)	Induced pluripotent stem cell(s)
MAPK	Mitogen-activated protein kinase
SA	Sinoatrial
VGCC(s)	Voltage-gated calcium channel(s)
